# Phospholipase A_2_ in Experimental Allergic Bronchitis: A Lesson from Mouse and Rat Models

**DOI:** 10.1371/journal.pone.0076641

**Published:** 2013-10-29

**Authors:** Rufayda Mruwat, Saul Yedgar, Iris Lavon, Amiram Ariel, Miron Krimsky, David Shoseyov

**Affiliations:** 1 Department of Biochemistry, Hebrew University Medical School, Jerusalem, Israel; 2 Department of Neurology, Hadassah University Hospital, Jerusalem, Israel; 3 Department of Biology, Faculty of Natural Sciences, University of Haifa, Haifa, Israel; 4 Pediatric Department, Hadassah University Hospital, Jerusalem, Israel; Virginia Tech University, United States of America

## Abstract

**Background:**

Phospholipases A_2_ (PLA_2_) hydrolyzes phospholipids, initiating the production of inflammatory lipid mediators. We have previously shown that in rats, sPLA_2_ and cPLA_2_ play opposing roles in the pathophysiology of ovalbumin (OVA)-induced experimental allergic bronchitis (OVA-EAB), an asthma model: Upon disease induction sPLA_2_ expression and production of the broncho-constricting CysLTs are elevated, whereas cPLA_2_ expression and the broncho-dilating PGE_2_ production are suppressed. These were reversed upon disease amelioration by treatment with an sPLA_2_ inhibitor. However, studies in mice reported the involvement of both sPLA_2_ and cPLA_2_ in EAB induction.

**Objectives:**

To examine the relevance of mouse and rat models to understanding asthma pathophysiology.

**Methods:**

OVA-EAB was induced in mice using the same methodology applied in rats. Disease and biochemical markers in mice were compared with those in rats.

**Results:**

As in rats, EAB in mice was associated with increased mRNA of sPLA_2_, specifically sPLA_2_gX, in the lungs, and production of the broncho-constricting eicosanoids CysLTs, PGD_2_ and TBX_2_ in bronchoalveolar lavage (BAL). In contrast, EAB in mice was associated also with elevated cPLA_2_ mRNA and PGE_2_ production. Yet, treatment with an sPLA_2_ inhibitor ameliorated the EAB concomitantly with reverting the expression of both cPLA_2_ and sPLA_2_, and eicosanoid production.

**Conclusions:**

In both mice and rats sPLA_2_ is pivotal in OVA-induced EAB. Yet, amelioration of asthma markers in mouse models, and human tissues, was observed also upon cPLA_2_ inhibition. It is plausible that airway conditions, involving multiple cell types and organs, require the combined action of more than one, essential, PLA_2_s.

## Introduction

Phospholipases A_2_ (PLA_2_) enzymes hydrolyze membrane phospholipids, producing arachidonic acid (AA). AA is metabolized into different lipid mediators, mainly through the cyclooxigenases (COXs), producing prostaglandins (PGs) and thromboxanes (TXs), and the lipoxygenases (LOs), producing leukotrienes (LTs) [1]–[3]. These include broncho-constricting ones, such as cysteinyl LTs, PGD_2_ and TXB_2_, as well as broncho-dilating ones, such as PGE_2_ [4], [5].

Accordingly, the control of PLA_2_ activities has been proposed for treating respiratory inflammatory/allergic diseases. Cellular PLA_2_s are generally classified into the intra-cellular cytosolic and the Ca^2+^-independent PLA_2_s (cPLA_2_s and iPLA_2_s, respectively), and the secretory PLA_2_s (sPLA_2_s). Previous studies have assigned a role for secretory and cytosolic PLA_2_s in inflammatory/allergic processes, while the iPLA_2_ does not seem to be significantly involved in airway pathology [6]–[10]. However, these studies have **not** produced an unequivocal conclusion.

In a previous study, we investigated the involvement of PLA_2_s and eicosanoids in asthma pathophysiology using a rat model of ovalbumin (OVA)-induced experimental allergic bronchitis (EAB) [4], [11], as expressed by broncho-constriction, airway remodeling, the levels of the broncho-dilator PGE_2_ and the broncho-constrictor Cysteinyl-LTs (CysLTs) in bronchoalveolar lavage (BAL). Upon induction of EAB these indices were up-regulated, except for PGE_2_ which was markedly reduced. Concomitantly, sPLA_2_ expression in lung tissue was enhanced, while cPLA_2_ expression was markedly decreased. All these parameters were reversed upon amelioration of the disease by treatment with an sPLA_2_ inhibitor, resulting in elevation of cPLA_2_ and PGE_2_ along with suppression of sPLA_2_ and Cys-LTs [4], [11].

PGE_2_, generally considered a pro-inflammatory mediator, is a potent broncho-dilator and inhibits smooth muscle cell proliferation [11]–[15]. It has thus been postulated that, unlike other organs, the lung is unique in benefiting from the action by PGE_2_ [11]. Therefore, the results obtained with the rat EAB model, seemed to make a clear physiological sense, suggesting that sPLA_2_ plays an important role in the onset and progression of asthma, while cPLA_2_ is involved in the disease abatement.

However, differing results were presented in studies with mouse models, mostly using PLA_2_ genetic manipulations. Henderson *et al.* [16], [17] assigned a key role to sPLA_2_gX, showing that physiological and biochemical markers of OVA-induced asthma were reduced in sPLA_2_gX -deficient mice [16]. These markers were enhanced when the mouse sPLA_2_gX was replaced with human sPLA_2_gX, or inhibited by treatment with a specific sPLA_2_gX inhibitor [17]. Munoz *et al.* [18] reported that cell migration and airway hyper-responsiveness were attenuated in OVA-sensitized PLA_2_gV-defficient mice, as well as by treatment of mice (WT) with sPLA_2_gV antibody. Similarly, Giannattasio *et al.* [19] showed that *Dermatophagoide farina*-induced lung inflammation was attenuated in sPLA_2_gV-deficient mice.

On the other hand, Uozomi *et al.* [20] showed that in cpla2-deficient mice OVA-induced anaphylactic response and bronchial reactivity to methacholine were significantly reduced. Similarly, Bickford *et al.* [21] showed that mice sensitized/stimulated with *Aspergillus fumigatus* exhibited marked elevation of cPLA_2_γ mRNA expression. These discrepancies might be due to differences in methodologies and/or genetic manipulations, or might reflect the involvement of more than one PLA_2_ type. To explore these possibilities, in the present study we examined the role of PLA_2_s in OVA-induced EAB **in mice**, without genetic manipulation of PLA_2_, using the **same methodology and procedures applied to rats** in our previous study [4], [11]. It was found that, similar to our findings with rats [4], [11], OVA-induced EAB in mice was associated with enhanced sPLA_2_ expression and production of broncho-constricting eicosanoids. However, in contrast to EAB in rats, cPLA_2_ mRNA expression and PGE_2_ production were elevated in the mouse model. Yet, in both models, the disease was markedly ameliorated by treatment with a cell-impermeable sPLA_2_ inhibitor.

## Materials and Methods

### Ethic statement

This study includes experiments with mice, all conducted according to the instruction and permit of the Hebrew University Ethical Committee

### Induction of experimental allergic bronchitis (EAB) in mice

As in our previous study in rats [4], [11], in the present study EAB was induced in BALB/c female mice by a weekly IP injection of 0.3 ml PBS containing 100 µg OVA, and 2 mg of the adjuvant Al(OH)_3_ for three weeks, followed with four weeks of challenge by three weekly intranasal (IN) OVA administration (100 µg in 50 µL PBS).

EAB development was assessed by two common tests:

1. **Pulmonary function.** by airway response to allergen or methacholine, using two non-invasive methods:
  - Enhanced pause (Penh): Unrestrained conscious mice were placed in a whole-body plethysmograph (Buxco Electronics, Troy, NY), measuring flow-derived pulmonary function (Penh), as previously described [4], [11], [17], [22].
  - Airway resistance using the occlusion technique (Roccl): Non-sedated mice, with closed mouth, were breathing through a nose-mask connected to a pneumotach (flow-meter) with a mouth pressure port. The pneumotach was attached to 2 differential pressure transducers, connected through preamplifiers (Hans Rudolph, Shawnee, KS, USA) producing analog signals of flow and mouth pressure, digitized by a data acquisition program (LabView National Instruments, Austin, TX). Peak pressure was measured while the mouse was breathing against an occluded pneumotach for 3–5 breaths. The pressures generated at the beginning and at the end of occlusion (inspiratory and expiratory, respectively) were divided by the respective adjacent peak flow immediately before and after the occlusion. Resistance (Rocclud) was calculated as peak pressure divided by the adjacent peak flow. Airway resistance is expressed as the percent change compared to baseline (level before treatment).Airway reactivity was assessed before challenge (baseline) and 5 minutes after IN challenge with either OVA or increasing methacholine dose (0, 40, 80, 320, 640, and 1280 µg in 20 µL PBS).
2. **Gene expression of arginase-I and mammalian acidic chitinase in lung tissue.** both enhanced in asthma. Arginase-I is involved in L-arginine metabolism and the subsequent inhibition of NO production, typical of type-2 responses [23], [24]. Although chitin does not exist in mammals, chitinases and chitinase-like proteins have been observed in mice and human subjects [25]. The prototypic acidic mammalian chitinase is induced during T_H_2 inflammation through an IL-13–dependent mechanism, and plays an important role in the pathogenesis of T_H_2 inflammation and IL-13 effector pathway activation [25]–[27]. The respective primers are depicted in **Table 1**.

**Table 1:** Primers sets for RT-PCR.

	*Sense*	*Anti-sense*
**18S**	5′TCGAGGCCCTGTAATTGGA 3′	5′CCCTCCAATGGATCCTCGTT 3′
**Arginase I**	5′TGAGCTCCAAGCCAAAGTCCT 3′	5′CAGCAGACCAGCTTTCCTCAGT 3′
**Acidic Chitinase**	5′CTGGTGAAGGAAATGCGTGAA 3′	5′ATGTTGGAAATCCCACCAGCT 3′
**PLA_2_gIVA**	5′CTTGTTCATTTTCGCCCACTTC 3′	5′CAGAGAGGTGTGGATCTTATCATC 3′
**PLA_2_gIVC**	5′TGCTGGTTTTGCCATCAACA 3′	5′GATTTCATGGCGTTGGCAGTA 3′
**PLA_2_gX**	5′CCACGTGACGCCATTGACT 3′	5′TGATGGTCCATGCACTTCCAT 3′
**PLA_2_gV**	5′CAAGGATGGCACTGATTGGTG 3′	5′GGTCCGAATGGCACAGTCTTT 3′

Broncho-alveolar lavage (BAL) was collected by lung washes (3×2 ml PBS), via tracheal cannulas, centrifuged to remove cells and kept at −80°C.

### Histological Analysis by Hematoxylin and Eosin Staining

Lungs preserved in 4% formaldehyde were dehydrated, sliced longitudinally, and embedded in paraffin. Histological sections of 4 µm thick were cut on a microtome, placed on glass slides, deparaffinized and stained sequentially with hematoxylin (for nuclear material) and eosin (for cytoplasmic material).

PLA_2_ mRNA expression in lung was determined by RT-PCR, using conventional methods [28]. Total RNA was purified from lung tissues (SV Total RNA isolation kit, containing DNase I Promega Corporation, Madison, WI) to remove possible genomic DNA contamination. RNA integrity was tested by 1% agarose gel electrophoresis. cDNA was prepared from total RNA (2 µg/ml) using MuLV reverse transcriptase (Applied Biosystems). Primers were designed using the Primer Express program (Applied Biosystems). Target mRNA was calculated in reference to the endogenous 18S ribosomal RNA, while the naive group was used as a calibrating factor. The respective primers are depicted in **Table 1**.

### Eicosanoids in BAL

Cysteinyl-LT (Cys-LT), PGE_2_, PGD_2_ and TXB_2_ were determined in BAL using ALIZA kits (Cayman Chemical, Michigan).

### 5-LO and 15-LO protein expression in lung was determined by Western blotting

Lung homogenate in lysis buffer [1% NP40, 0.5% sodium-deoxycholate, 0.1% sodium- dodecyl-sulfate, 2 mm EDTA, 50 mm NaF, 0.2 mm orthovanadate and protease inhibitor cocktail, in PBS pH 7.2], were centrifuged (20000 *g* for 15 min) and the supernatant protein content was determined (Bradford Reagent, Sigma). 20 µg protein (boiled in 1×SDS sample buffer) was separated by SDS–10% polyacrylamide gel electrophoresis (PAGE) and blotted with rabbit-anti-mouse 5- or 15-LO antibodies in 5% BSA in TBST (for 18 h at 4°C), followed by incubation with the appropriate secondary antibody (horseradish peroxidase-conjugated to goat anti-rabbit antibody). The membranes were washed (3× TBST, 5 min each) before and after incubation (1 h, 20°C), and visualized by chemiluminescence (West Pico, Pierce, Rockford, IL), as described [29].

### Treatment with cell-impermeable sPLA_2_ inhibitor

As in the previous study of EAB in rats [4], [11], we have tested the effect of a cell-impermeable sPLA_2_ inhibitor, composed of PLA_2_-inhibiting lipid (specifically derivatized phosphatidyl ethanolamine), conjugated to hyaluronic acid (HyPE), which prevents the inhibitor's internalization, thereby designed to confine the inhibitory action to the cell membrane. This inhibitor has been shown to suppress the action of exogenous sPLA_2_s and diverse related inflammatory conditions in a number of studies [4], [11], [30]. The mice were treated during the challenge, one hour before each OVA challenge, with IN administration of HyPE (200 µg in 50 µl at the first two challenges, followed by 40 µg in 40 µl, until one day before sacrifice).

Statistical analysis was done using one-way ANOVA, followed by Tukey multiple comparison. Conventionally, *P*≤0.05 was considered significant.

## Results

### Induction of OVA-induced EAB in mice

**Figure 1:**
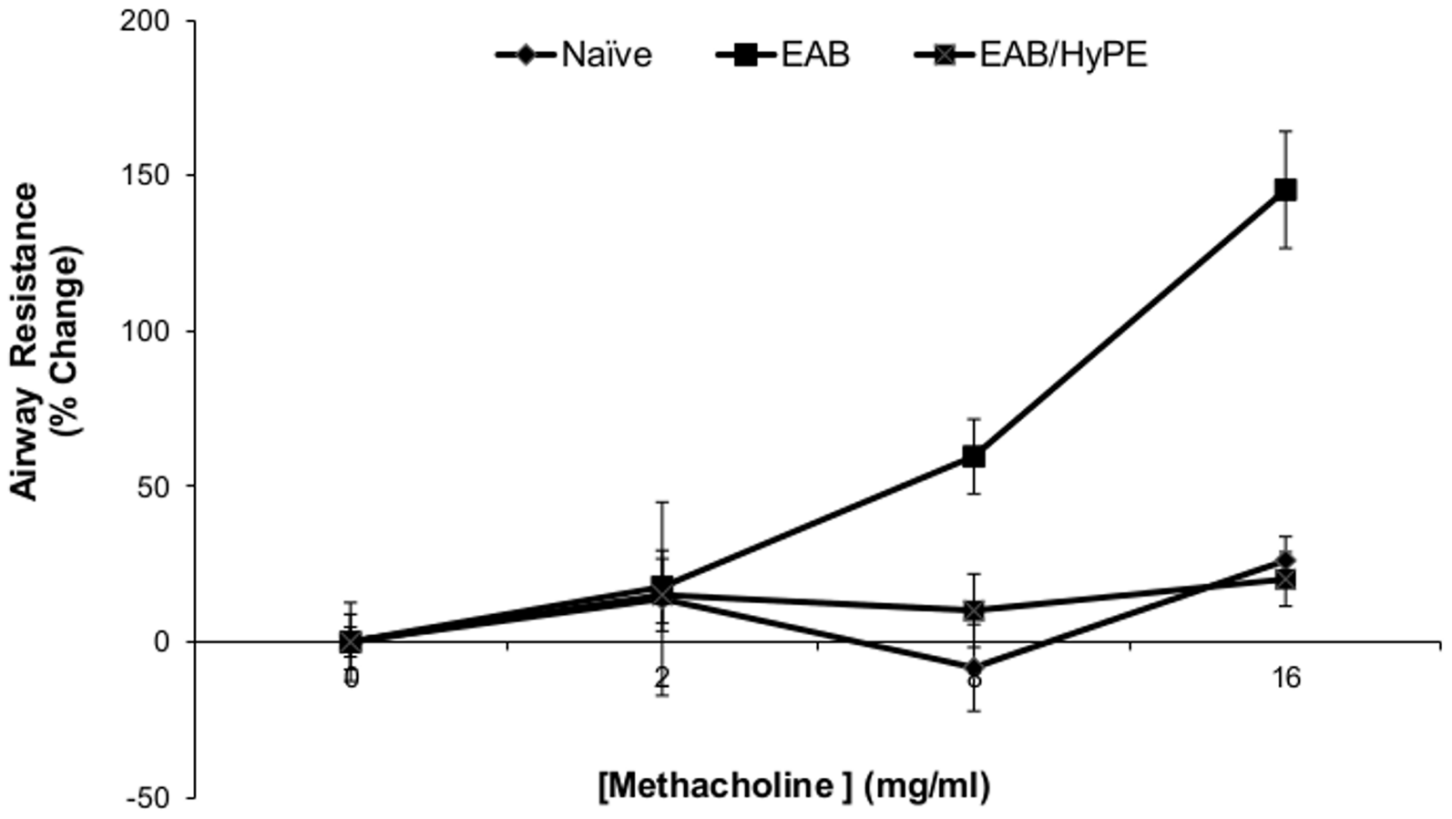
Airway resistance of EAB mice following methacholine challenge. Mice with OVA-induced EAB (EAB-Mice), with/without treatment with sPLA_2_ inhibitor (EAB and EAB/HyPE, respectively), were challenged with methacholine. Airway resistance was determined as described in Methods. Data are mean ± SEM for 8 mice. *, *P*<0.01 for the highest dose.

**Figure 2:**
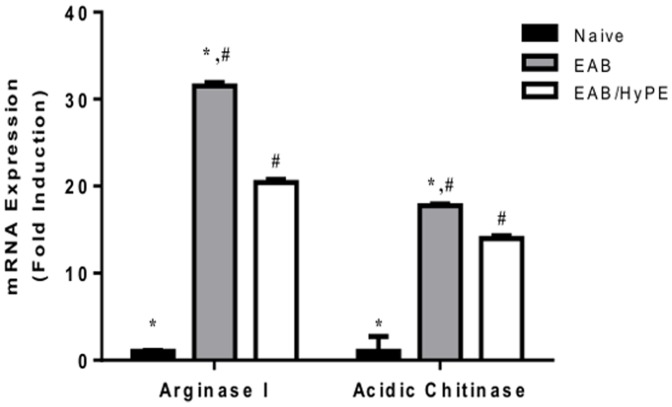
mRNA expression of arginase- I and acidic chitinase in lungs of EAB mice. mRNA of arginase-I and acidic chitinase in lungs was determined by RT-PCR. Each datum is mean ± SEM for 10 mice in a group. *, # *P*<0.05.

**Figs. 1** **&** **2** demonstrate the validation of the EAB induction, showing that methacholine challenge exerted airway resistance to air flow in a dose-dependent manner (**Fig. 1**), concomitantly with enhanced expression of arginase-I and chitinase mRNA (**Fig. 2**). Similar to our findings with rats [4], [11], the elevation of these physiological and biochemical markers was inhibited by treatment with the sPLA_2_ inhibitor.

### Airway response to OVA challenge

**Figure 3:**
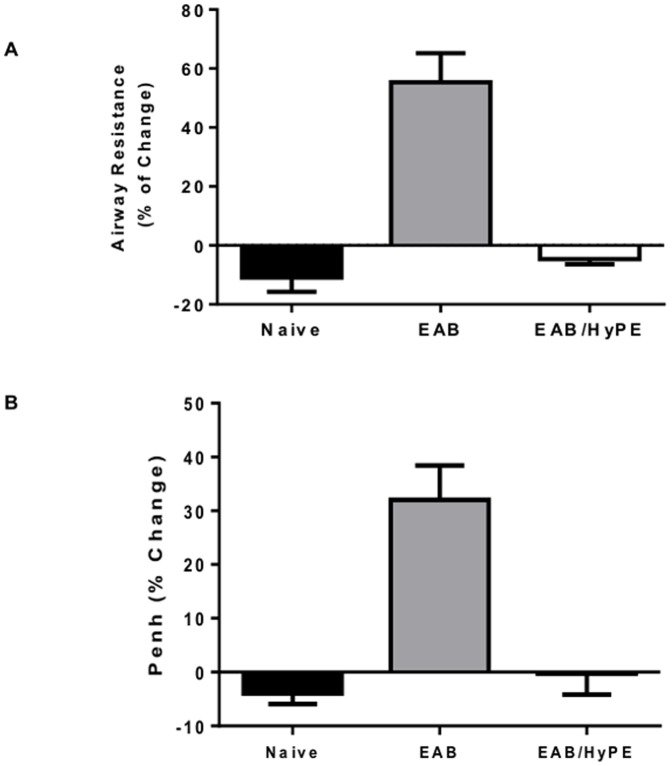
Airway response to challenge in mice with OVA-induced EAB. Mice were subjected to OVA challenge and airway response was determined by airway resistance (3A) and Pulmonary enhancement (Penh, 3B), as described in Methods. In 3A, data are mean ± SEM for 8 mice, *, # *P*<0.05. In 3B, data are mean ± SEM for 10 mice, *, #, *P*<0.01.

**Figure 4:**
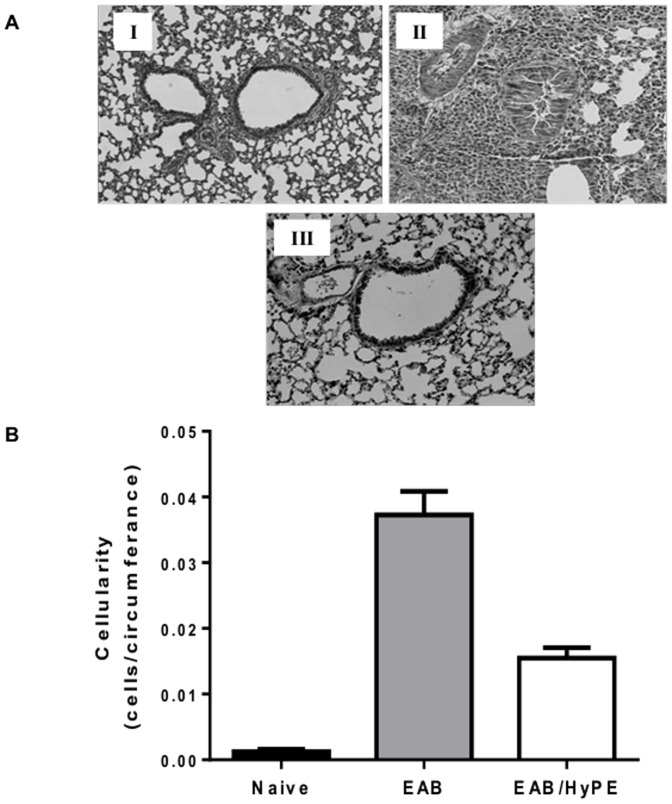
Lung histology of mice with OVA-induced EAB. A. Representative micrographs of lung histology: Mice lung tissues were stained with hematoxylin and eosin. I: Healthy mice II: Untreated EAB mice. III: EAB mice treated with HyPE. B. Peri-bronchial infiltration of inflammatory cells. The number of leukocytes in lung peri-bronchial space was determined by morphometry. Data are mean ± SEM for 10 mice.*, #, *P*<0.01.

Mice with OVA-induced EAB responded to OVA challenge with markedly enhanced airway resistance, as expressed both by Penh (**Fig. 3A**) and resistance (**Fig. 3B**). Similarly, EAB induction was associated with peribronchial infiltration of inflammatory cells, as shown in the histology micrographs (Fig. 4A) and in the respective morphometric measurement (**Fig. 4B**). These figures also show that pre-treatment with the sPLA_2_ inhibitor completely prevented the disease development, reverting both the airway response (**Figs. 3A &3B**) and the inflammatory cell infiltration (**Figs. 4A & 4B**), to their level in naïve mice.

### PLA_2_s expression in lungs

**Figure 5:**
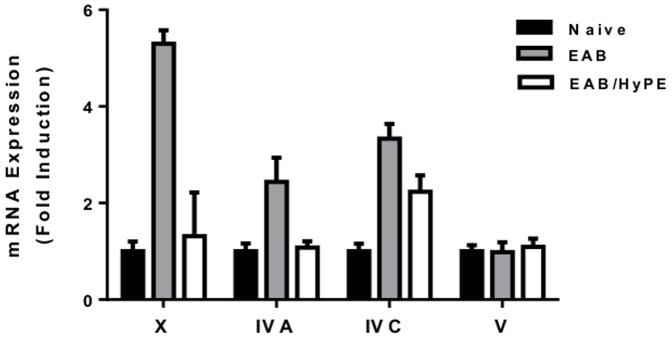
mRNA expression of PLA_2_s in EAB mice lung. mRNA of PLA_2_s in mice lung homogenates was determined by RT-PCR. Each datum is mean ± SEM for 10 mice in a group. Significant difference between naïve and EAB (P<0.01), and between EAB and EAB/HyPE (P<0.05) was found for sPLA_2_gX and for cPLA_2_gIVC. No significant difference was found for sPLA_2_gV.

As noted above, in the rat model the disease induction was associated with suppression of cPLA_2_ expression [4], [11], while studies with mice suggested that the disease induction involved elevated expression of PLA_2_gIVC (cPLA_2_γ) [21] RNA expression, as well as sPLA_2_gV [18], [19] and sPLA_2_gX [16], [17]. In the present study we have found that while sPLA_2_gV, sPLA_2_g1B and sPLA_2_gIII were not affected by the disease induction, the expression of both sPLA_2_gX and cPLA_2_γ was markedly increased, and both were suppressed by treatment with the sPLA_2_ inhibitor (**Fig. 5**). The elevated sPLA_2_ expression is in agreement with our findings in the rat model [4], [11], and with other studies in mice [16]–[18]. However, the elevated cPLA_2_ expression, while in agreement with others' studies with mice [20], [21], is in contrast to our findings in rats, where cPLA_2_ was suppressed in the disease state, and resumed upon treatment with the sPLA_2_ inhibitor.

### Eicosanoids in BAL

**Figure 6:**
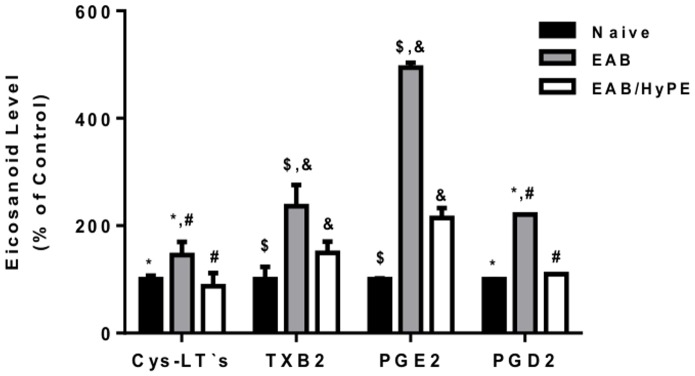
Eicosanoid level in BAL of EAB mice. Eicosanoids in the mice BAL were determined by ELISA. Results are percent change relative to control (100%). The absolute control levels (100%) were 51.47 pg/ml for Cys-LTs, 101.83 ng/ml for TXB_2_, 7.85 ng/ml for PGE_2_ and 378.11 pg/ml for PGD_2_. Data are mean ± SEM for 10 mice. *, #, *P*<0.05; $, &, *P*<0.01.

**Fig. 6** shows that, in parallel to the PLA_2_ expression, EAB induction was associated with enhanced production of both the broncho-constricting PGD_2_, TXB_2_ and CysLTs, and the broncho-dilating PGE_2_, which is in agreement with previous studies with mice [4], [11].The elevation of the broncho-constricting eicosanoids in the disease state is in accordance with our findings in the rat model [4], [11]. However, the elevated PGE_2_ production observed here is in contrast to our findings in the rat model, where the disease induction was associated with suppression of PGE_2_.

### Expression of 5-lipoxygenase

**Figure 7:**
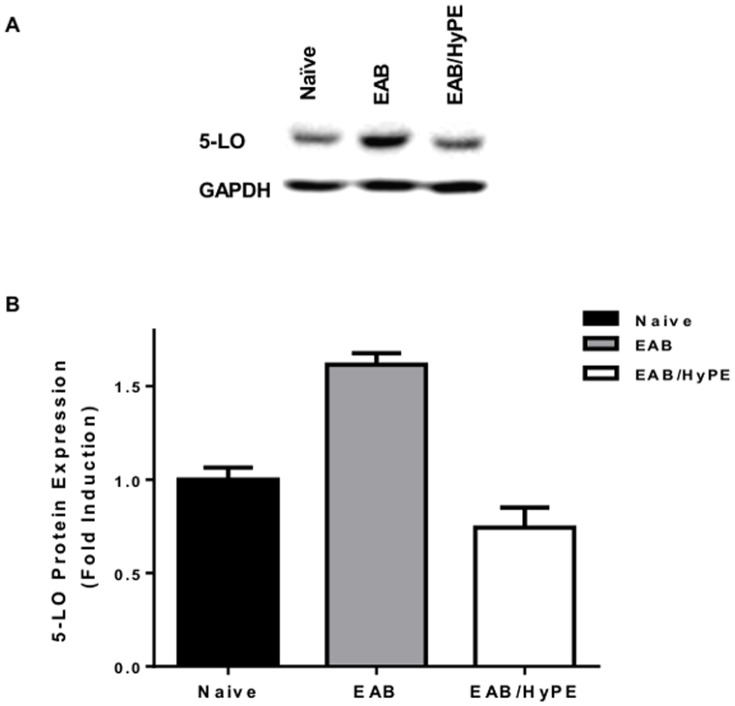
5-LO protein level in EAB mice lung. 5-LO protein in mice lung homogenates was determined by Western blotting A. Representative blots. B. Blot quantification by densitometry, normalized to GAPDH. Data are mean ± SEM for 3 independent experiments, normalized to GAPDH. Data are mean ± SEM for 3 independent experiments. * P<0.05.

In recent years, airway inflammation has been shown to undergo temporal changes from the inflammatory phase, where 5-lipoxygenase (5-LO) produces the broncho-constricting LTs, to a resolution phase, in which 15-LO produces anti-inflammatory lipid mediators, such as protectins and resolvins [31]–[35]. The EAB model applied in the present study does not reach the resolution phase. Accordingly, as shown in **Fig. 7**, the EAB induction was associated with elevation of 5-LO protein expression, which was suppressed by treatment with the sPLA_2_ inhibitor, whereas 15-LO expression was not affected by the disease or its treatment (not shown).

## Discussion

### PLA_2_ expression

As discussed in the Introduction, previous studies with mouse models of asthma have produced differing results, showing that the disease was associated with increased expression of sPLA_2_gX [16], [17], sPLA_2_gV [18], [19], or cPLA_2_ [20], [21], and was ameliorated by treatment with specific inhibitors or genetic manipulations of these enzymes. Our previous study with the rat EAB model [4], [11] conforms to the studies with mice, pointing to sPLA_2_s as a key player in asthma pathophysiology [16], [17]. However, in contrast to the previous studies with mice that associated the disease with elevated cPLA_2_ expression [20], [21], in rats we have found that the disease was associated with suppression of cPLA_2_ expression. To determine whether these discrepancies reflect differences between species or methodologies (e.g., genetic manipulation, stimulants, selection of PLA_2_ isoforms studied), in the present study we applied to mice, with no genetic manipulation, the same protocol of OVA-induced EAB used in the rat study [4], [11]. The results of both models, summarized in **Table 2** show that, similar to the findings with rats, OVA-induced EAB in mice was associated with increased sPLA_2_gX, conforming to the finding of Henderson *et al.* [16], [17], while sPLA_2_gV was not affected. However, contrary to our findings with rats, where cPLA_2_ expression was suppressed in the disease state, OVA-induced EAB in mice was associated with elevated expression of cPLA_2_γ and cPLA_2_α, which agrees with previous mouse studies assigning a role for cPLA_2_ in asthma pathophysiology [20], [21]. In addition, Giannattasio *et al.* [36] reported that IgG-stimulated human lung mast cells are a source for several sPLA_2_s that contribute to LTC_4_ production, known to facilitate asthma development. Subsequently, in the present study we also examined mRNA expression of some of the reported sPLA_2_ isoforms, specifically sPLA_2_gXIIA, sPLA_2_gXIIB, sPLA_2_gIB, sPLA_2_gIII, and sPLA_2_gVI in the mice lung, and found that none of them was affected in the OVA-induced EAB (not shown). It therefore seems that the results would differ between animal models, depending on the species and methodologies used.

**Table 2:** Physiological and biochemical markers of OVA-induced EAB in rat^1^ and mouse^2^.

	EAB		EAB+sPLA_2_ inhibitor	
	Rat	Mouse	Rat	Mouse
Pulmonary Resistance	↑	↑	↓	↓
sPLA_2_ expression	↑	↑	↓	↓
CysLT	↑	↑	↓	↓
cPLA_2_ expression	↓↓	↑↑	↑↑	↓↓
PGE_2_	↓↓	↑↑	↑↑	↓↓

1 Data retrieved from our previous studies of OVA-EAB in rats [4], [11].

2 Data of current study.

Another limitation of the OVA-induced EAB in mice, and possibly of the other models discussed above, is indicated by the finding that EAB is associated with elevation of 5-LO (Fig. 7), known to be involved in the disease induction, whereas 15-LO, which involved in the disease resolution [33], was not affected (**data not shown**). This might suggest that these animal models reflect different phases of the course of the disease. It is not unlikely that PLA_2_ expression varies at different phases and this contributes to the discrepancies between the expressions of PLA_2_ isoforms observed in the various studies with animal models.

### Lipid mediators

As shown in **Table 2** the induction of EAB in rats was associated with suppressed production of PGE_2_, concomitantly with enhanced production of Cys-LT, and both were reversed upon disease amelioration [4], [11]. This is physiologically sound, since PGE_2_ is a broncho-dilator, and Cys-LTs is a broncho-constrictor [37]. However, OVA-induced EAB in mice is associated with elevation of both the broncho-dilator PGE_2_ and the bronco-constricting eicosanoids, Cys-LTs [4], TBX_2_ and PGD_2_. This is in agreement with the above–discussed studies reporting that in the mouse asthma model the disease state is characterized by elevated production of both types of eicosanoids [16], [20], and these were inhibited, along with the other disease indices, by inhibition of either sPLA_2_ or cPLA_2_.

Notably, in the study with a mixed human lung cell population, cPLA_2_ inhibition decreased the ionomycin-induced production of PGD_2_, LTB_4_ and TXA_2_, but not that of PGE_2_ [37]. Since PGE_2_ is a broncho-dilator [4], the authors considered that as a positive outcome of the treatment. In line with that, in the present study, the treatment with an sPLA_2_ inhibitor strongly suppressed, practically to the basal (naïve) level, the elevated production of CysLTs, TXB_2_ and PGD_2_, while PGE_2_ level was only partially reduced (Fig. 6), thereby turning their balance toward the broncho-dilating PGE_2_. This supports the notion that airway pathophysiology is ultimately determined by the balance between the dilating and constricting lipid mediators.

It should be noted that the research on inflammatory lipid mediators in airway conditions has addressed predominantly the eicosanoids. However, PLA_2_ activity is also responsible for the production of lyso-phospholipids, some of which are known to be potent inflammatory/allergic mediators; e.g. lyso-phosphatidyl-serine activates mast cells to secret histamine, lyso-phosphatidic acid induces muscle cell proliferation, and lyso-phosphatidyl-choline is the precursor of PAF [4], [38] and more [39]. Therefore, the focus on eicosanoids might provide only part of the picture, as it ignores the potentially major role of lyso-phospholipids and the respective PLA_2_ activities in airway pathophysiology.

### Use of PLA_2_ inhibitors

An intriguing phenomenon presented by the present and previous studies on PLA_2_ in asthma-related pathophysiology in mouse and rat models, is that the disease was successfully treated by specific inhibitors or genetic manipulations of specific PLA_2_s, including sPLA_2_gX, sPLA_2_gV and cPLA_2_γ [16]–[21].

Similarly, the study of Hewson *et al.* [37] showed that a specific inhibitor of cPLA_2_α inhibited the contarctility of AMP-stimulated isolated human tarcheal rings, as well as eicosanoids production by mixed human lung cells and IgE-stimulated mast cells. On the other hand, in a recent study (Mruwat et al., unpublished), we have found that the production of inflammatory/allergic cytokines (IL-5, IL-13, IL-17 and INF-γ) by cultured human nasal polyps stimulated with super antigen, was associated with increased expression of sPLA_2_gX, and suppression of cPLA_2_α expression. Yet, both cytokine production and PLA_2_ expression were reversed by treatment with the sPLA_2_ inhibitor used in the present study.

Taken together, the studies with animal models and human tissues discussed above, appear to suggest that since several tissues and cell types take part in the pathophysiology of asthma and related airway conditions [9], [36], it is plausible that the disease development requires a combined (likely sequential) action of more than one essential PLA_2_ - from different cell types - and blocking one of them would significantly attenuate the disease. This hypothesis conforms to the model of Murakami *et al.* [40], proposing various modes of cross-talk between sPLA_2_ and cPLA_2_ in the induction of airway diseases.

In conclusion, the findings and considerations summarized above demonstrate that animal models can provide only limited insight into the role of PLA_2_ isoenzymes in the pathophysiology of human airway diseases. As these conditions involve multicellular/multi-organ processes, it is plausible to conclude that human asthma and related conditions require the combined action of more than one essential PLA_2_ isoform. By changing the ratio between the pro-and anti-inflammatory lipid mediators - eicosanoids and lyso-phospholipids - PLA_2_ inhibition would determine the disease resolution. Which PLA_2_ isoform(s) should be the target for pharmacological inhibition is yet to be explored and will ultimately be decided based on comprehensive clinical studies.
